# Ciliary and Non-Ciliary Roles of IFT88 in Development and Diseases

**DOI:** 10.3390/ijms26052110

**Published:** 2025-02-27

**Authors:** Xuexue Wang, Guoyu Yin, Yaru Yang, Xiaoyu Tian

**Affiliations:** Center for Cell Structure and Function, Shandong Provincial Key Laboratory of Animal Resistance Biology, College of Life Sciences, Shandong Normal University, Jinan 250014, China; 17353655845@163.com (X.W.); yin13026556091@163.com (G.Y.); yaru1229@163.com (Y.Y.)

**Keywords:** cilium, ciliopathy, IFT88, IFT, ORPK mouse model

## Abstract

Cilia are highly specialized cellular projections emanating from the cell surface, whose defects contribute to a spectrum of diseases collectively known as ciliopathies. Intraflagellar transport protein 88 (IFT88) is a crucial component of the intraflagellar transport-B (IFT-B) subcomplex, a protein complex integral to ciliary transport. The absence of IFT88 disrupts the formation of ciliary structures; thus, animal models with IFT88 mutations, including the oak ridge polycystic kidney (ORPK) mouse model and IFT88 conditional allelic mouse model, are frequently employed in molecular and clinical studies of ciliary functions and ciliopathies. IFT88 plays a pivotal role in a variety of cilium-related processes, including organ fibrosis and cyst formation, metabolic regulation, chondrocyte development, and neurological functions. Moreover, IFT88 also exhibits cilium-independent functions, such as spindle orientation, planar cell polarity establishment, and actin organization. A deeper understanding of the biological events and molecular mechanisms mediated by IFT88 is anticipated to advance the development of diagnostic and therapeutic strategies for related diseases.

## 1. Introduction

### 1.1. Cilia and Intraflagellar Transport (IFT) Complex

Cilia represent highly specialized cellular appendages that protrude from the cellular surface and are ubiquitous across a wide range of organisms, from unicellular eukaryotes to vertebrates. In vertebrates, cilia are categorized into motile and non-motile types based on their structural and functional attributes [1]. Non-motile cilia, also termed primary cilia, serve crucial roles in chemical and osmotic sensation, phototransduction, and the regulation of signaling pathways [2]. Notably, several signaling cascades, including the Wnt, Hedgehog (Hh), and mechanical pathways, are modulated by primary cilia [3].

During cilia biogenesis and structural maintenance, the tubulin heterodimers comprising the ciliary axoneme maintain a dynamic equilibrium characterized by continuous assembly and depolymerization at the ciliary tip [4,5,6,7,8]. During cilium formation, microtubule precursors enter the cilium and are transported along the axoneme to the assembly site at the ciliary apex. Conversely, during cilia depolymerization, the depolymerized tubulin heterodimers are transported back to the ciliary base and recycled within the cell body [9]. This intricate process necessitates the presence of an intraflagellar transport (IFT) mechanism, which facilitates the continuous shuttling of proteins between the ciliary base and tip via the IFT complex [8,10].

IFT is a sophisticated and tightly regulated microtubule-based transport system that facilitates the movement of proteins along the ciliary axoneme [11]. It functions as a protein carrier by binding both cargo proteins and motor proteins, thereby orchestrating their transport between the ciliary base and tip [12]. Various proteins, including microtubule-associated proteins, membrane receptors, and signal transduction factors, have been reported to be transported by IFT [12,13]. The IFT particles consist of IFT-A and IFT-B subcomplexes, comprising at least 6 and 16 distinct polypeptides, respectively. The binding of the IFT-B subcomplex with kinesin-2 mediates the anterograde transport of ciliary proteins, while the binding of the IFT-A subcomplex with dynein facilitates retrograde transport [14]. Two types of motor proteins interact with cargo either directly or through associated factors such as the BBSome, IDA3, ODA16, ARMC2, and TULP family proteins [15]. IFT trains, consisting of two protofilaments assembled from IFT particles, enable multiple interactions between particles and ensure the stability of the transport sequence. Each IFT train comprises approximately 1000 protein subunits, assembled from IFT particles and motors, and spans between 100 and 700 nanometers in length [13,15].

Ciliary microtubules are extensively decorated with a variety of post-translational modifications (PTMs), which exert a significant influence on IFTs. For example, it was reported that the tubulin glutamate ligase gene *ttll-*4 becomes activated by the p38 mitogen-activated protein kinase (MAPK) pathway, thereby inducing glutamylation of tubulin within sensory ciliated axons in *Caenorhabditis elegans* [16]. The glutamylation of tubulin serves as a crucial determinant of IFT efficiency, with a direct and positive correlation observed between the operational efficacy of IFT and the degree of tubulin glutamylation [16]. It was also revealed in *Chlamydomonas* that when tubulin undergoes ubiquitination, IFT139 (or other constituent subunits of the IFT-A complex) engages in interactions with ubiquitylated α-tubulin or a broader spectrum of ubiquitylated proteins, facilitating their transport towards the cell body [17].

IFT88 is a pivotal member of the IFT-B subcomplex [18]. Cells harboring mutations in IFT88 exhibit a notable reduction in the abundance of IFT57 relative to other IFT proteins, suggesting that IFT88 plays a crucial role in the assembly of specific IFT particles [19]. It was discovered that IFT88 is crucial for the formation of primary cilia in embryonic nodes, kidneys, limbs, and other tissues during mammalian development [19,20], and the absence of IFT88 completely abrogates the formation of ciliary structures [18,21,22]. Given the significant functional roles of IFT88 in both ciliary and non-ciliary processes, we will elucidate the functions, molecular mechanisms, and clinical applications of animal models pertaining to development and related diseases, offering insights for future research endeavors.

### 1.2. Structure and Expression of IFT88

#### 1.2.1. Molecular Structure of IFT88

IFT88 serves as a pivotal component within the IFT-B complex, bridging the IFT-B1 and IFT-B2 subcomplexes and localizing at the cilium’s tip [23,24]. It plays a crucial role in maintaining the bidirectional movement of tubulin heterodimers along axonemes, which is indispensable for the formation and functionality of primary cilia [9,19,25,26].

**Figure 1:**
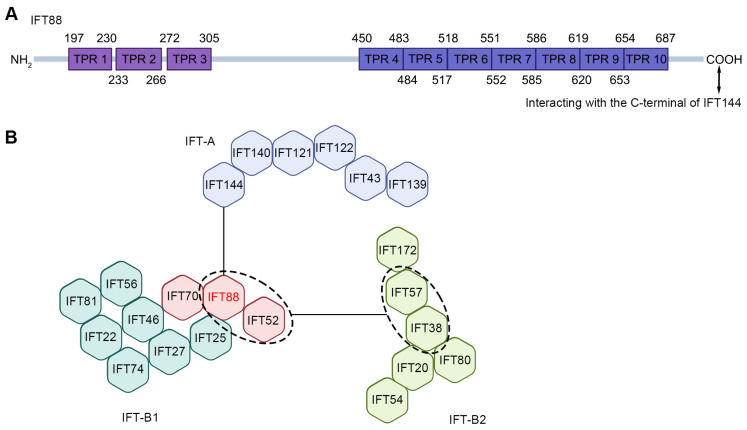
Structure of IFT88. (**A**) The topological structure of IFT88. (**B**) Components and interactomes of the IFT-A and IFT-B complexes. The position of IFT88 is highlighted.

Intramolecular cross-linking analysis reveals a periodic structure in IFT88, characterized by a 34–50-residue repetition, indicative of the presence of tetrapeptide repeat (TPR) domains (Figure 1A). These domains consist of concise arrays of 34-amino acid sequences and are instrumental in protein–protein interactions. Specifically, the amino half of IFT88 harbors three contiguous TPR domains, while the carboxyl half contains seven additional domains [19]. Aside from these 10 TPRs, no other discernible motifs have been identified in IFT88 [19,27]. No identifiable patterns have been found in the sequence of IFT88, except for in its comparison with the Protein Data Bank (PDB), which reveals significant similarity to the N-acetylglucosaminyltransferase (OGT) members. Both proteins feature numerous TPRs arranged into an elongated superhelix, with the concave side lined by conserved asparagines crucial for recognizing binding partners. The conservation of these asparagines in the IFT88 sequence suggests a comparable target recognition mechanism [19].

Within the IFT protein complex, IFT88 adopts a loose and open superhelical conformation [27]. The interaction between the C-terminal of IFT88 in the IFT-B1 subcomplex and the C-terminal of IFT144 in the IFT-A subcomplex facilitates the association between IFT-A and IFT-B (Figure 1A,B) [28]. Although these elements are positioned close enough for interaction, the structural rigidity necessary for a tight connection in the anterograde train is lacking due to the long and disordered nature of the IFT88 C-terminal. Consequently, the interaction between IFT88 and IFT144 can be regarded as the initial contact in a multi-step recruitment process, where a loose initial attachment precedes a tighter binding to achieve a mature anterograde structure [15,28,29].

In IFT-B1, IFT88, IFT70, and IFT52 exist in the form of IFT88/70/52 trimers. IFT52 and IFT88 form the main interface between IFT-B1 and IFT-B2. This is mediated through interactions with IFT57/38 of IFT-B2. Conserved proline residues in IFT57 and IFT38 create a right-angled kink that points the subsequent coiled-coil segment toward IFT88 in the same repeat. The loose spiral of IFT88 creates an open cleft, which IFT57/38 and the IFT52 disordered region slot into, creating multiple contacts between the IFT-B1 and IFT-B2 components (Figure 1B) [28].

#### 1.2.2. Cellular Expression of IFT88

During mammalian development, IFT88 is expressed in both ciliated and some non-ciliated cells [19,30].

As the core of the IFT-B complex among IFT proteins, IFT88 is indispensable for cilium formation [18]. IFT88 deficiency disrupts cilium assembly, resulting in the absence of cilia in cells [18,19]. Many vertebrate tissues possess non-motile primary cilia, such as embryonic lymph nodes, kidneys, limbs, nerves, and cartilage [18,19,31]. Primary cilia protrude from the surface of cells and receive signals from the environment [32]. These signals are integrated to provide outputs required for cell proliferation, differentiation, migration, and polarization [32]. For instance, the Hh signaling pathway is a vital ciliary signaling mechanism involved in tissue patterning, organogenesis, and tumorigenesis, with IFT88 playing a significant role in regulating this pathway [32,33,34].

Beyond its ciliary functions, IFT88 is also expressed in non-ciliated cells and fulfills non-ciliary roles [35]. For example, it has been reported to control directed cell division during zebrafish protocorm formation, independently of cilia [30].

The centrosome constitutes a pivotal element within the tubulin-based cytoskeleton. In addition to its roles in constructing the intracellular microtubule network and forming the mitotic spindles, centrosomes are also integral to the positioning of cilia and flagella [36,37]. In both ciliated and non-ciliated proliferating cells, IFT88 maintains a close association with centrosomes throughout the cell cycle [38]. Its assembly on centrosomes is independent of microtubules and dynamin and directly influences cellular progression. Variations in IFT88 concentration levels impact the progression of the G1/S cell cycle [38]. Cells expressing IFT88 that are arrested before the S phase undergo apoptosis. Conversely, silencing IFT88 promotes cell cycle progression into the S and G2/M phases [38].

IFT88 exhibits functional conservation across the entire eukaryotic phylogenetic tree. Homologs of IFT88 have been identified in diverse organisms, encompassing mammals, *Drosophila*, nematodes [21], ciliates [39], trypanosomes [40], and fish [41]. Interestingly, in the unicellular parasite *Trypanosoma cruzi*, the depletion of IFT88 does not impact the structural integrity of fully developed flagella but rather disrupts their beating motion. This finding hints at a potential dysregulation or mislocalization of flagellar components that are integral to flagellar motility, such as PKAR, kinesin-9, and FAM8 [40].

## 2. Functional Roles of IFT88 in Disease and Development

### 2.1. Cilium-Related Functions of IFT88

**Figure 2:**
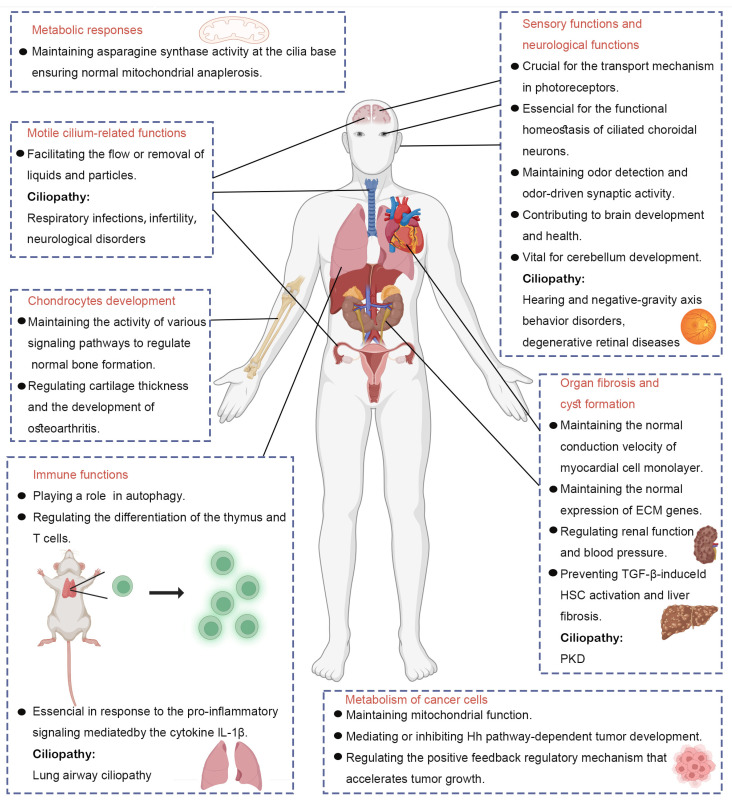
Cilium-related functions of IFT88. IFT88 is essential for the proper formation and function of primary cilia in multiple organs and is implicated as a key contributor to various ciliopathy phenotypes.

Cilia are ubiquitous in cells and tissues, serving crucial physiological functions. The absence of IFT88 during development can impair ciliary signaling and lead to severe developmental defects [42]. In humans, alterations in cilium formation and function can disrupt organ function and manifest clinically as ciliopathies, a diverse set of disorders characterized by defects in cilium assembly, maintenance, and signaling [43,44]. Polycystic kidney disease (PKD), retinitis pigmentosa (RP), and Leber congenital amaurosis (LCA) are among the most extensively studied ciliopathies [32]. Furthermore, various recessive ciliary syndromes affecting multiple organs with varying severity, including the kidneys, liver, eyes, ears, brain, bones, and reproductive system, have been identified (Figure 2) [32].

IFT88 has been implicated as a key contributor to various ciliopathy phenotypes [23]. Studies have shown that reduced cilia numbers are associated with lower IFT88 levels [19,45]. As a crucial constituent within the IFT-B complex, IFT88 plays an important role in facilitating the interconnection between IFT-B1 and IFT-B2, as well as bridging the interaction between IFT-A and IFT-B [28]. As an important component of the IFT complex, IFT88 is essential for the bidirectional movement of tubulins along axonemes and the normal formation of primary cilia [19,26].

#### 2.1.1. Motile Cilium-Related Functions

Motile cilia facilitate the flow or removal of liquids and particles through coordinated whip-like beats [46]. Epithelial cells in the respiratory tract, fallopian tubes, and ventricles of the brain contain motile cilia [32,46]. Additionally, the flagellum plays a pivotal role in spermatozoon motility. Defects in motile cilia can result in respiratory infections, infertility, and neurological disorders [32,46].

Mice with motor cilia defects, caused by the deletion of the *Ift*88 gene (also called *tg*737, which is a homolog of the ciliated protein OSM5 in *Caenorhabditis elegans* and the flagellate component IFT88 in *Chlamydomonas Rhine*) [47], exhibit growth retardation and severe hydrocephalus, leading to an enlarged skull and increased cranial pressure [48]. Mutations in IFT88 cause the loss of motile cilia in blood vessels, potentially impairing blood flow and resulting in a pathology similar to obstructive hydrocephalus [48].

#### 2.1.2. Organ Fibrosis and Cyst Formation

Disruptions in primary cilium functions are associated with fibrosis in various tissues, such as the heart, liver, and kidneys [49,50]. Primary cilia have been identified as regulatory factors for fibroblast activation and extracellular matrix (ECM) deposition [49]. In a study, a short interfering RNA (siRNA) knockdown approach was employed to decrease the expression of the IFT88 gene to eliminate primary cilia from fibroblasts derived from rat ventricles, enabling the observation of the subsequent effects on the ECM and conduction in a co-culture system comprising cardiomyocytes (CMs) and FBs. The results revealed that the knockdown of IFT88 significantly upregulated the expression of ECM genes, namely *Ctgf*, *Fn1*, and *Col1a1* [51]. These findings suggest that IFT88 plays a pivotal role in regulating cilia, thereby maintaining the normal expression levels of *Ctgf*, *Fn1*, and *Col1a1*, and preventing their overexpression.

Reduced expression of IFT88, which is required for ciliary assembly in fibroblasts in the heart, disrupts primary cilium formation and affects cardiomyocytes [52]. This can lead to increased atrial fibrosis, ECM remodeling, and abnormal cardiomyocyte conduction [53,54]. Atrial fibrosis promotes the development and persistence of atrial fibrillation by facilitating re-entry [54]. IFT88 maintains the normal conduction velocity of myocardial cell monolayers through cilia and prevents arrhythmias [51].

Recent research has shown that primary cilia are present on quiescent hepatic stellate cells (HSCs) and undergo significant loss upon HSC activation, promoting liver fibrosis.

This correlates with decreased levels of IFT88 [55,56]. Liver fibrosis is characterized as excessive deposition of the ECM in the liver, in response to chronic liver injury induced by various factors. Persistent liver fibrosis often progresses to cirrhosis and hepatocellular carcinoma, representing significant global contributors to morbidity and mortality [57,58]. *Ift88*-knockout (KO) mice are more susceptible to chronic carbon tetrachloride-induced liver fibrosis. X-linked inhibitor of apoptosis (XIAP) acts as an E3 ubiquitin ligase for IFT88. Transforming growth factor-β (TGF-β), a pro-fibrotic factor, enhances XIAP-mediated ubiquitination of IFT88 and promotes its degradation by the proteasome [59,60,61,62]. The XIAP-IFT88 axis was identified as a potential therapeutic target for liver fibrosis. Blocking XIAP-mediated IFT88 degradation prevents TGF-β-induced HSC activation and liver fibrosis [57].

Nephron-specific IFT88 promotes primary cilium formation in the nephron and regulates renal function and blood pressure [63,64]. Primary cilia maintain the balance of mRNA involved in ECM synthesis and degradation, as well as the levels of fatty acid β-oxidation-related factors, including 3-hydroxybutyrate, medium-chain acyl-CoA dehydrogenase, and acylcarnitine. IFT88 mutation causes primary ciliary loss and PKD in mice. Previous studies have linked cyst formation to ciliary dysfunction. In the kidney, cilia function as mechanical organelles tasked with detecting fluid flow through the tubular lumen [65]. Numerous studies have corroborated their role in sensing the movement of fluid within the renal tubular lumen. The impairment of this mechanosensory function is postulated to be a crucial determinant in the pathogenesis of cyst formation [66]. In zebrafish, both IFT88 mutants and IFT172 mutants exhibited the formation of cysts, indicating that mutations in ciliary proteins resulted in ciliary dysfunction, ultimately leading to the development of renal cysts [67]. In the oak ridge polycystic kidney (ORPK) mouse model of PKD, ciliary dysfunction is caused by a mutation in a sub-state of IFT88, rather than a deletion of the gene [43,68]. Relative changes in fatty acid β-oxidation, renal ECM metabolism, and other pathways precede cystogenesis in IFT88 KO mice [64,69]. Furthermore, gender-related disparities in the performance of these aspects have been identified [64].

#### 2.1.3. Metabolic Responses

Nutrient deprivation triggers an energy crisis that requires metabolic and organelle reorganization for resolution. Primary cilia play a critical role in sensing nutrient availability [70]. They can detect glutamine levels and regulate their length during metabolic stress through asparagine synthase-facilitated glutamine-mediated anaplerosis, thereby facilitating the cell’s response to glutamine [71,72,73,74]. Nutrient deprivation leads to ciliary elongation, which is mediated by reduced AMPK activation, ATP availability, and mitochondrial function [75,76]. IFT88 maintains cilia presence and asparagine synthase expression and activity at the cilia base during metabolic stress, ensuring normal glutamine-dependent mitochondrial anaplerosis [77].

#### 2.1.4. Metabolism of Cancer Cells

Primary cilia are present in most mammalian cells and are involved in Hh signal transduction during development. As unique signaling organelles, primary cilia can mediate or inhibit Hh pathway-dependent tumor development through IFT88, depending on the nature of the carcinogenic initiation events [78].

Cancer cells are frequently associated with the impairment of primary cilia and IFT functions [79]. The mutation causing the functional loss of IFT88 leads to substantial impairments in ciliogenesis and mitochondrial oxidative function [79,80]. The gene expression profile of IFT88-deficient thyroid cancer cells favors glycolysis and lipid biosynthesis [79]. However, the absence of IFT88/primary cilia does not augment the proliferation, migration, and invasion of thyroid cancer cells [78,79]. In thyroid cancer cells, IFT88 has been implicated in metabolic reprogramming, but not in its tumor-suppressive capacity [79].

Cancer cells and osteoblasts exhibit a positive feedback regulatory mechanism that accelerates tumor growth [3]. Osteoblasts secrete TNF-α to inhibit cancer cell proliferation, a mechanism regulated by osteoblasts’ IFT88. Cancer cells, in turn, secrete TGF-β to suppress the expression of the IFT88 gene in osteoblasts, thereby disrupting this inhibitory mechanism. Consequently, as cancer cells proliferate, their inhibitory effect on bone cells diminishes [3]. This signaling interplay between bone cells and cancer cells is prevalent in breast and prostate cancer, suggesting a potential therapeutic target for mitigating bone tumor growth in cancer patients [3].

Additionally, the functions of IFT88 in cancer may also be associated with its non-ciliary-related roles, including the regulation of cell division (through spindle orientation and modulation of the cell cycle), the control of cell migration, and the establishment of immune synapses in lymphocytes. Therefore, the ciliary and non-ciliary functions of IFT88 are often difficult to distinguish and require a comprehensive and dialectical analysis.

#### 2.1.5. Chondrocyte Development

IFT88 is pivotal for somatic development. Mutations in IFT88 cause developmental anomalies such as polydactyly and abnormal neural tube patterns [12]. During endochondral bone formation, IFT88 maintains the activity of various signaling pathways, including the Sonic hedgehog (Shh) and Indian hedgehog (Ihh) pathways, to regulate normal bone formation in limbs [12]. The IFT88 protein plays a crucial role in regulating cartilage thickness and the development of osteoarthritis in mice, functioning as a positive regulator of cartilage thickness [81]. In mouse models of osteoarthritis, IFT88 preserves the Hh signaling threshold during physiological loading to regulate cartilage calcification, thereby protecting articular cartilage [81]. The absence of IFT88 in cartilage leads to a decrease in cilia in the growth plate, disrupting chondrocyte differentiation, cartilage resorption, and mineralization [82]. Inhibiting IFT88 reduces hypertrophic chondrocyte vascular endothelial growth factor (VEGF) expression, vascular recruitment, osteoclast activity, and cartilage replacement [82].

#### 2.1.6. Craniofacial Complex Development

The development of the craniofacial complex is a highly orchestrated process that necessitates intricate interactions among tissues derived from diverse embryonic origins. Any disruption in this process can impede the normal formation of the face and culminate in severe craniofacial anomalies [83,84]. Non-syndromic cleft lip with or without cleft palate (NSCLP) is a prevalent craniofacial anomaly in humans. Prior research has demonstrated that the loss of functional primary cilia significantly impacts the developing craniofacial complex, leading to a spectrum of anomalies, including premature cranial suture closure, midface hypoplasia, dental anomalies, and NSCLP [85]. IFT88, a vital protein for ciliogenesis, has emerged as a candidate gene for NSCLP [84]. A study focusing on NSCLP in non-Hispanic white and Hispanic families revealed that two noncoding intronic variants (rs9509311 and rs2497490) in IFT88 were positively associated with NSCLP in the Hispanic white population, suggesting potential race-specific effects [86].

A separate study of patients with non-syndromic cleft lip and cleft palate identified missense mutations in the IFT88 coding sequence, which may represent a partial loss of IFT88 function, ultimately causing a ciliopathy associated with human cleft palate [84]. This mutation was located within the third of TPR structural domains. These domains are thought to serve as scaffolds, facilitating protein–protein interactions and the assembly of multiprotein complexes [84].

#### 2.1.7. Sensory Functions and Neurological Functions

In *Drosophila melanogaster* [87], DmIFT88 maintains sensory function by positioning signaling proteins along *Drosophila* cilia [88]. DmIFT88 is crucial for the ciliary localization of DmGucy2d (*Drosophila* Guanylyl Cyclase 2d). IFT88 is essential for ciliary sensory function in sensory neurons, partly through the binding and localization of various signaling proteins, such as Iav and DmGucy2d [88]. The acute loss of DmIFT88 in sensory neurons subtly alters the ciliary curvature at the base without affecting the ultrastructure of axon filaments, impairing ciliary sensory function and leading to hearing and negative-gravity axis behavioral disorders [87,88,89]. DmIFT88 is also essential for the functional homeostasis of ciliated choroidal neurons, and mutations in DmIFT88 can cause degenerative retinal diseases [87,88]. At the same time, IFT52 plays an essential role in sensory cilium formation and neuronal sensory function in *Drosophila* [90]. It can be seen that cilia are crucial to the sensory function of neurons.

Beyond *Drosophila*, IFT88 plays a significant role in the photoreceptors of vertebrates. Vertebrates require the daily transfer of large amounts of lipids and proteins from the inner segment (IS) to the outer segment (OS) of photoreceptors [91]. Defects in intra-photoreceptor transport lead to retinopathy. Vertebrate photoreceptors are polarized sensory neurons composed of a photosensitive OS that develops from primary cilia [92]. IFT is crucial for the transport mechanism in several animal photoreceptors, and dysfunction may cause retinal degeneration, such as LCA and RP [23,91,92]. IFT88, as an IFT protein particle, has been reported to localize in photoreceptors, particularly at the IS-OS boundary where cilia are located [91]. Mice with IFT88 mutations develop an abnormal OS, leading to progressive photoreceptor degeneration. *Zebrafish* lacking IFT88 exhibited a complete absence of an OS but retained some ISs, whereas zebrafish deficient in IFT172 lacked both an OS and IS [67]. These observations underscore that distinct ciliary protein deletions impact cilia to varying degrees, with subsequent effects on photoreceptors.

The cilia of olfactory sensory neurons (OSNs) serve as primary sites for odorant binding, and their loss results in anosmia. In *Ift*88^osnKO^ mice, the absence of OSN cilia led to a substantial decline in odor detection and odor-driven synaptic activity within the olfactory bulb. Notably, the restoration of wild-type IFT88 in these mice rescued OSN ciliation and olfactory function [93]. Furthermore, IFT88 mutants displayed consistently reduced and selective responses of OSNs to bile acids and food due to specific ciliogenesis defects [94].

IFT88 has been shown to facilitate the formation of normal cilia in the brain, thereby contributing to the maintenance of healthy neurological functions in adults, including learning, memory, and debris removal [95,96]. In one study, forebrain-specifc IFT88 KO mice exhibited severe learning disabilities in fear-conditioned reflex and Morris water maze tests. Additionally, IFT88 KO mice displayed altered sleep patterns and reduced phase–amplitude coupling, a crucial process underlying learning and memory formation [95]. This study underscores the pivotal role of primary cilia in learning and memory functions [95]. Microglia, phagocytes responsible for synaptic pruning and debris removal during brain development and homeostasis, were also implicated. In Alzheimer’s disease, suppressing IFT88 expression in microglia altered their behavior, leading to the enlargement of extracellular amyloid plaques and damage to adjacent synapses [96]. The knockdown of IFT88 affected extracellular vesicle-mediated secretion of β-amyloid (Aβ) and promoted the accumulation of axonospheres. The ectopic accumulation of extracellular vesicles at neuronal axon terminals triggered neuronal atrophy in Alzheimer’s disease [96].

The essential role of IFT88 in cilium formation also influences brain development and damage repair. The knockdown of *Ift88* resulted in the deletion of cilia in oligodendrocyte precursor cells, leading to decreased proliferation, which is crucial for promoting development and proliferation in white matter injury [97]. *Wnt1-Cre; Ift88^flox/flox^* mutant embryos exhibited malformed heads, enlarged forebrains, and severely hypoplastic olfactory bulbs at embryonic day 18.5 (E18.5) [98]. In another study, researchers utilized a subnormal allele of IFT88, termed *cobblestone*, to demonstrate the critical function of primary cilia in dorsal telencephalic development. *Cobblestone* mutants displayed severe forebrain regionalization defects, characterized by dorsomedial telencephalic disorganization, encompassing the choroid plexus, cortical hemispheres, and hippocampus [99]. Mice harboring *Ift88* mutations exhibit neural tube closure and pattern defects [22], which studies have demonstrated to be attributable to impaired Shh signaling [12]. Additionally, given that the cell cycle also influences brain development, the cilium-independent functions of IFT88 in cell cycle regulation, described below, may also play a role in brain development. However, the specific mechanisms involved require further investigation.

IFT88 is vital for the normal development of the cerebellum, and defects in granulosa cell proliferation are central to the cerebellar pathology observed in human cilium-related diseases. The disruption of the IFT88 gene results in severe cerebellar hypoplasia associated with the proliferation failure of granular progenitor cells in the external granular layer (EGL) [100]. Granulocyte progenitor cell proliferation is sensitive to partial loss of IFT function in IFT88 hypomorphic mutants (IFT88 ORPK), and this effect is modulated by the genetic background [100].

#### 2.1.8. Immune Functions

Lung airway ciliopathy is characterized by an elevated cytokine level and a diminished proportion of anti-inflammatory T regulatory cells [101]. The deletion of the IFT88 gene in adult mice results in the absence of lung airway epithelial cilia and the subsequent development of bronchiectasis, ultimately progressing to lung airway ciliopathy [101,102,103]. Furthermore, primary cilia play a role in autophagy, which is anti-inflammatory [104]. The interplay between primary cilia and cellular autophagy mechanisms [97] offers compelling evidence to support the hypothesis that the protein encoded by IFT88 possesses certain immune functions [101].

Additionally, IFT88 in thymic epithelial cells regulates the differentiation of both the thymus and T cells. In IFT88 KO mice lacking primary cilia in thymic epithelial cells, there is a notable increase in CD4^+^ and CD8^+^ single-positive thymocyte subsets, accompanied by mild disorganization of the intercellular contact between T cells and the “thymic synapse” of medullary thymic epithelial cells [105]. The depletion of cilia in crucial organs through IFT88 deletion reduces the expression of Programmed cell death 1 ligand 1 (PD-L1), resulting in uncontrolled local T-cell proliferation and activation, which may underlie ciliopathic phenotypes [106]. IFT88 is essential for the differentiation of double-positive thymocytes into single-positive thymocytes, and IFT88-sufficient T cells possess the capability to compensate for the functional deficiencies of impaired cells, thereby occupying the available niche [107].

Primary ciliary transport is essential in response to the pro-inflammatory signaling mediated by the cytokine interleukin-1β (IL-1β) [108]. In the chondrocytes, mutations in IFT88 lead to the loss of cilia, impeding primary ciliary transport and inhibiting the associated pro-inflammatory resistance response [108]. The mechanical inhibition of the inflammatory response of chondrocytes to IL-1β partially occurs through an IFT-dependent pathway facilitated by HDAC6 activation and tubulin acetylation and polymerization [109]. Recent studies have further elucidated the interaction between pro-inflammatory nuclear factor-kappa B signaling and IFT [110].

Apart from its cilium-associated roles, the modulation of immune function by IFT88 encompasses cilium-independent functions, specifically pertaining to the formation of lymphocyte immune synapses, as elaborated below.

### 2.2. Cilium-Independent Functions of IFT88

**Figure 3:**
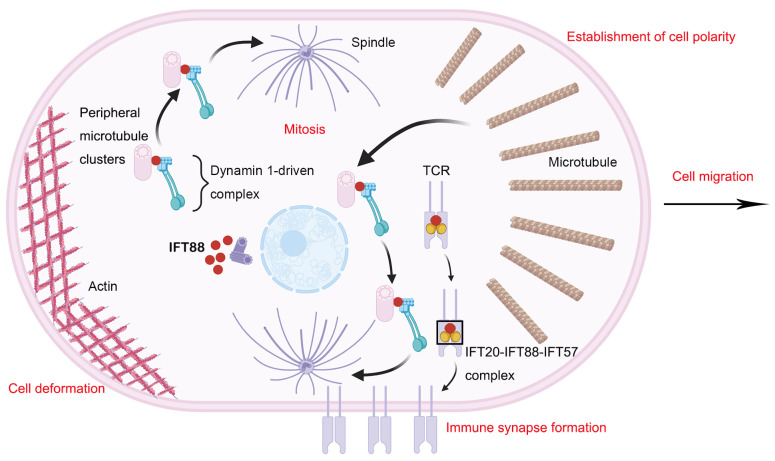
Cilium-independent functions of IFT88. IFT88 plays crucial roles in cell division, cell cycle progression, and spindle orientation. It functions as an integral component within a dynamin 1-driven complex, which is responsible for transporting peripheral microtubule clusters, containing microtubule nucleating proteins, to the spindle pole. This process facilitates the formation of astral microtubule arrays. Furthermore, cells deficient in IFT88 exhibit a reduced number of microtubules at the leading edge. Additionally, IFT88 regulates the organization of basal actin, resulting in alterations in cell deformation and mechanical properties.

In addition to its cilium-related functions, IFT88 has also been reported to exhibit cilium-independent functions (Figure 3) [47]. A complex and reciprocal relationship exists between IFT and cytoskeletal structures, potentially influencing fundamental cell behavior.

#### 2.2.1. Cell Migration

The establishment of cell polarity is indispensable for cell migration. Microtubules play a crucial role in the establishment of cellular polarity [111,112]. Cells deficient in IFT88 exhibit a reduction in microtubules at the leading edge [47]. Microtubules primarily originate at the microtubule-organizing center (MTOC) and additional sites, including the Golgi. In migrating cells, a subset of microtubules extend into the leading edge, delivering proteins that affect the protein composition, microtubule behavior, the actin–myosin network, and vesicles at the leading edge [47]. In IFT88-depleted cells, the number of Golgi orientations within ±60° perpendicular to the leading front angle is significantly decreased. The absence of IFT88 disrupts the polarization of migrating cells, thereby impairing cell migration. In summary, IFT88 promotes cell migration in a cilium-independent manner by fostering directional polarity and altering the microtubule cytoskeleton (Figure 3) [47].

#### 2.2.2. Mitosis

Due to their degradation prior to mitotic entry, cilia are absent during the mitotic phase of the cell cycle [113]. Previous research has indicated that IFT88 fulfills a cilium-independent function during mitosis [30], specifically impacting microtubules [113].

The orientation of the mitotic spindle determines the direction of cell division [114]. During mitosis, IFT88 plays a pivotal role in organizing and orienting the spindle (Figure 3) [30,47,113]. It is also essential for the formation of stellate microtubules [47,113]. Cytoplasmic dynamin 1 contributes to the IFT88-dependent spindle pole localization of microtubules. IFT88 operates as part of a dynamin 1-driven complex in mitotic cells, which transports microtubule nucleating protein-containing peripheral microtubule clusters to the spindle pole and facilitates the formation of astral microtubule arrays [113]. The absence of IFT88 leads to significant loss and shortening of astral microtubules, ultimately causing spindle misalignment [47,113].

IFT88 is indispensable for the accurate accumulation of NuMA at the minus ends of k-fibers during mitosis, and it interacts with NuMA in mitotic extracts to re-anchor it to the spindle by regulating NuMA accumulation [115]. NuMA has been demonstrated to play a pivotal role in promoting spindle assembly and maintaining the integrity of its constituent factors [116]. In the event of spindle integrity disruption, IFT88 swiftly identifies the minus ends of k-fibers and initiates an efficient re-anchoring response by facilitating the appropriate accumulation of NuMA [115]. This proficient repair mechanism aids in preserving spindle integrity, thereby ensuring the spindle’s resilience to perturbations and facilitating the effective alignment of chromosomes [116].

Furthermore, IFT88 influences the G1/S transition during cell division [47]. Centrosomes serve as the organizing centers of the cytoskeleton and play a crucial role in most microtubule-dependent processes [117,118], including the mediation of cytokinesis. As a centrosomal protein, IFT88 regulates the G1/S transition in non-ciliated cells. IFT88 remains tightly associated with the centrosome throughout the cell cycle in a microtubule- and dynein-independent manner [38]. In G1- or G2-phase cells, IFT88 is present in perinuclear focal points co-located with γ-tubulin, showing enrichment similar to γ-tubulin. During the G1 phase, IFT88 is located at the proximal ends of maternal centrioles. In the early S phase, centrioles begin to duplicate, and duplication is usually completed by G2/M. At the G2/M transition, IFT88 is localized at the proximal ends of both mother and daughter centrioles. By metaphase, when centrosomes mature, IFT88 is situated within the centrosome [38]. Centrosomes serve as nucleation and organizing sites for cytoplasmic microtubules [117,118]. Overexpression of IFT88 inhibits the cellular G1/S transition and induces apoptotic cell death, while IFT88 silencing promotes cell cycle progression into the S, G2, and M phases. A reduction in IFT88 expression facilitates entry into the S phase, leading to increased proliferation rates in these cells [38]. Cilia never form during this process.

#### 2.2.3. Actin Organization

Mutations in IFT88 have been shown to affect actin organization in various cell types [119,120]. IFT88 regulates the organization of basal actin and actin cortical stiffness, resulting in alterations in cell deformation and mechanical properties [121]. During cartilage development, the loss of IFT88 results in changes in the in situ actin tissue, decreased actin cortical formation, and reduced mechanical properties of chondrocytes [121,122]. These changes impact the cell’s ability to deform, equilibrium modulus, and bubble formation.

#### 2.2.4. Immune Synapse Formation

In lymphocytes, IFT88 is part of the endocytotic cycle targeting T-cell receptors (TCRs) to immune synapses, a process in which cilia never form [123]. The IFT20-IFT88-IFT57 complex exists in lymphocytes and plays a role in immune synapse formation and T-cell activation. Each component of the complex can interact with the TCR/CD3 complex. Continuous TCR signaling is required for T-cell activation [124]. Polarization of recirculating endosome-localized TCR/CD3 complexes into immune synapses driven by the microtubule-organizing center (MTOC) is crucial for mobilizing new receptors to this location [125]. The IFT20-IFT88-IFT57 complex promotes the formation of functional immune synapses by regulating the TCR/CD3 cycle. In response to signals triggered by surface TCRs, IFT20, IFT88, and IFT57 work together to deliver circulating endosome-localized TCR/CD3 to immune synapses as the MTOC polarizes towards the contact site with antigen-presenting cells (APCs) (Figure 3) [123,126].

IFT88 exhibits both cilium-related and cilium-independent functions. However, given the extensive association of cilia with numerous pathways, and the intricate involvement of various physiological and biochemical responses and metabolic pathways in each disease, it can be challenging to definitively delineate cilium-related functions from cilium-independent ones. To establish a clearer distinction between these functions, further meticulous and detailed studies are warranted.

## 3. Animal Models and Clinical Applications

Initially, primary cilia were deemed of minimal significance to human health, yet a growing body of evidence has implicated ciliary defects in numerous human diseases [19,127,128]. IFT88, a crucial protein for cilium formation, renders the investigation of IFT88 mutant animal models a vital approach to understanding human ciliopathies (Table 1) [9,19,25,26]. However, homozygous null mutations in IFT88 (IFT88^tm1Rpw^) result in embryonic lethality during early organogenesis [23,43]. Consequently, scientists frequently utilize ORPK mice harboring IFT88 hypomorphs, IFT88 conditional allelic mice, and ENU mutagenesis mice as alternative IFT88 mutant animal models [19,127,128,129,130]. These models offer profound insights into the mechanisms by which cilia influence cell behavior and how ciliary dysfunction disrupts tissue physiology, ultimately paving the way for potential therapeutic interventions in ciliopathies [12,131].

### 3.1. ORPK Mouse Model

#### 3.1.1. Characteristics of ORPK Mouse Model

The ORPK mouse stands as a seminal animal model for ciliopathy, cystic kidney disease, and ciliary dysfunction, establishing a pivotal link between cystic kidney disease and ciliary dysfunction [19,127,128]. Unlike the embryonically lethal IFT88 null mutations (*Ift*88^tm1Rpw^), the hypomorphic nature of the IFT88 allele in ORPK mice permits these homozygous mutants to survive into young adulthood [23,43]. Consequently, the ORPK mouse has emerged as an exemplary model for elucidating the role of primary cilia in diverse tissues [131].

ORPK mice were generated through the integration of a transgene into an intron adjacent to the 3′ end of the IFT88 gene, resulting in a hypomorphic allele (*Ift*88^Tg737Rpw^) [127,131]. The *Ift*88^Tg737Rpw^ mutation partially disrupts the expression and function of the IFT88 protein, thereby disrupting IFT, the necessary process for the assembly of both motile and non-motile cilia. The destruction of IFT leads to ciliary dysplasia and deformity, albeit not complete ablation [127,131].

**Figure 4:**
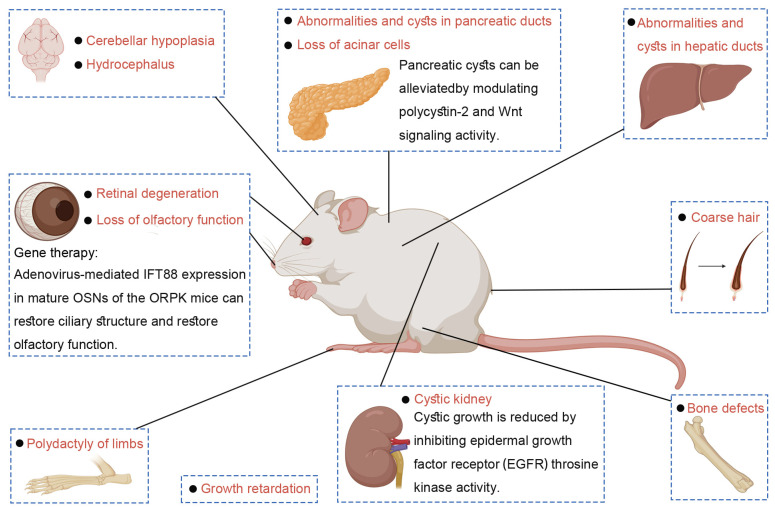
Characteristics and clinical applications of the ORPK mouse model. The ORPK mouse model exhibits a series of phenotypes, including coarse hair, growth retardation, polydactyly of the limbs, cystic kidney, cysts in hepatic and pancreatic ducts, cerebellar hypoplasia, hydrocephalus, bone defects, and retinal degeneration. Therapeutic targeting to these phenotypes serves as a valuable tool for analyzing the role of primary cilia in various tissues and organs.

The ORPK mouse model exhibits distinct phenotypes (Figure 4) characterized by coarse hair, severe growth retardation, and polydactyly of the limbs [136]. Notably, the most prominent phenotype observed in ORPK mice is their cystic kidney phenotype, which mimics human autosomal recessive polycystic kidney disease (ARPKD) [136]. Beyond cystic nephropathy, histological analyses of ORPK mice have also revealed abnormalities and cysts in hepatic and pancreatic ducts, cerebellar hypoplasia, hydrocephalus, bone defects, and retinal degeneration [48,91,100,132,136,137].

#### 3.1.2. Applications and Clinical Perspectives of ORPK Mouse Model

The ORPK mouse has been extensively utilized as a model organism in investigating the pathogenesis and treatment of ciliopathies (Figure 4), thereby significantly advancing the scientific and medical understanding of ciliary functions and ciliopathic conditions [12].

Although previous research has made substantial strides in identifying the genetic causes of ciliopathies, effective treatments for these disorders remain elusive [138,139]. Notably, ORPK mice exhibit functional anosmia due to the absence of cilia on their OSNs [138,139]. In an ORPK mouse model, a groundbreaking approach involving gene therapy was developed to address ciliary defects and restore olfactory function. Specifically, adenovirus-mediated expression of IFT88 in mature, fully differentiated OSNs of ORPK mice was sufficient to rejuvenate the ciliary structure and olfactory function [43,138,139]. This study represents the first instance of in vivo ciliary reconstruction in a mammalian model of ciliopathies, suggesting that gene therapy holds promise as a viable treatment option for repairing the ciliary structure and function in differentiated cells [43].

Furthermore, ORPK mouse models of polycystic kidney disease (PKD) have been instrumental in elucidating the crucial role of primary cilia in pancreatic tissue [132]. Approximately 10% of patients with autosomal dominant polycystic kidney disease (ADPKD) develop pancreatic cysts [140,141]. To gain insights into the mechanisms underlying pancreatic abnormalities in PKD, a study was conducted to analyze the pancreas formation and maturation in ORPK mice [132]. The findings revealed characteristic pancreatic defects in ORPK mice, including extensive acinar cell loss, abnormal tubular structures, and the presence of endocrine cells within the ducts [132,140,141]. These pancreatic cells in ORPK mice exhibited a reduced cilia number and an abnormal cilia structure, suggesting that inappropriate cilium assembly underlies the pancreatic defects. Additionally, polycystin-2, a protein implicated in PKD, was misexpressed in ORPK mice. There were alterations in the cellular localization of beta-catenin, a protein involved in cell adhesion and Wnt signaling, leading to deregulated Wnt signaling activity [132]. Thus, pancreatic PKD phenotypes are at least in part mediated through the deregulation of Wnt signaling activity [132]. Pancreatic cysts can be alleviated by modulating polycystin-2 and Wnt signaling activity.

ORPK mouse models have also been employed to demonstrate the involvement of Na^+^/H^+^ exchanger (NHE) activation in epidermal growth factor (EGF)-induced renal cyst formation. EGF plays a pivotal role in renal development [142], renal cyst formation [143], and renal metabolism [144]. In ORPK mouse models, EGF promotes the mitosis of collector duct cells by enhancing the exchange activity of NHE on the cell surface, thereby inducing renal cyst formation [133,145]. This cystic growth can be mitigated by inhibiting epidermal growth factor receptor (EGFR) tyrosine kinase activity [146,147].

The ORPK mouse model serves as a valuable tool for analyzing the role of primary cilia in various tissues and organs, significantly aiding in the assessment of the molecular, cellular, and physiological connections between ciliary dysfunction and disease pathogenesis in human ciliopathies [131]. It is noteworthy that allelic variation is prevalent in human ciliopathies, as exemplified by Joubert syndrome, pyelitis, and Meckel syndrome. Despite their diverse phenotypes, mutations in shared genes have been identified as potential causes of several forms of nephronophthisis (NPH), Meckel–Gruber syndrome (MKS), and Joubert syndrome (JBS), with phenotypic outcomes being contingent upon the nature of the mutation [148,149]. Genetic analysis utilizing inbred ORPK mice from diverse backgrounds will provide crucial insights into potential modifier genes associated with ciliary dysfunction [148,149]. The ORPK mouse model holds promise in the design of drug therapeutic targets and is anticipated to make a substantial contribution to the treatment of human ciliopathies [131].

### 3.2. IFT88 Conditional Allelic Mouse Model

#### 3.2.1. Characteristics of IFT88 Conditional Allelic Mouse Model

Mutations in the IFT88 gene result in the absence of cilia and lead to embryonic lethality during mid-gestation stages [22,150]. The premature demise of IFT88 mutants has impeded investigations into the role of cilia in later developmental phases [22,150]. These mutations also induce developmental abnormalities, including abnormal neural tube patterns and polydactyly. To address the role of cilia in limb development, researchers developed the IFT88 conditional allele [12]. Utilizing the Cre-lox system, it is feasible to eliminate cilia from various cell populations within the developing limb. Haycraft et al. engineered an IFT88 allele, *Ift*88*^fl^*, which contains 4–6 loxP sites flanking its exon, enabling the disruption of IFT88 through cre-mediated recombination [12]. Excision of these exons causes a translational frameshift, resulting in the loss of all IFT88 functions [12].

The col3.6cre mouse strain is frequently employed in studies of bone formation and has been previously described as “osteoblast-targeted cre” [129]. Cre recombinases are actually active in a variety of other cell types. Cre activity is found throughout the embryo, including the anterior intestinal endoderm, dorsal aortic wall, and neural tube [129,130]. The presence of Cre activity can be confirmed by eGFP expression. Therefore, other organs can also be studied by constructing conditional allelic mouse models of other organs. In the heart, eGFP is expressed in the myocardium, endocardium, epicardium, and posterior pSHF near the pulmonary endoderm [129,130]. Consequently, a new model of cilium-related cardiovascular abnormalities is developed in mice. By conditionally deleting IFT88 from various tissues, researchers can elucidate the role of cilia in cardiovascular development [129,130].

#### 3.2.2. Applications and Clinical Perspectives of IFT88 Conditional Allelic Mouse Model

The IFT88 conditional allelic mouse model has been instrumental in studying the role of cilia in limb development [12,81,151]. While the deletion of cilia in the limb ectodermal region did not significantly alter finger patterns, interstitial disruption led to severe polydactyly deformities, loss of anterior and posterior finger patterns, and shortening of both proximal and distal axes [12]. Conditional mutants exhibited defects in endochondral bone formation. Analysis of Ihh pathway expression in these mutants through radioactive in situ hybridization revealed a decrease in Ihh expression. This decrease was evident in all skeletal elements of the developing limbs, suggesting that IFT88 is crucial for Ihh signaling during embryonic endochondral bone formation [12]. Abnormal finger patterns were associated with abnormal Shh pathway activity, while growth defects in the limbs were partially attributed to disrupted Ihh signaling during endochondral bone formation. Thus, IFT88 plays a pivotal role in the normal formation of limb bones by regulating multiple signaling pathways [12,81,151].

IFT88 conditional allelic mice serve as valuable models for investigating the regulation of primary cilia in enamel formation [152]. Patients with ciliopathies frequently exhibit anomalies in tooth enamel. Kudo et al. employed mice with an epithelial-specific deficiency in *Ift*88 (*Ift*88*^fl/fl^*;K14 Cre) to explore the impact of primary cilia on enamel formation. Their study revealed that *Ift*88*^fl/fl^*;K14 Cre mice exhibited premature abrasion of their molars. Notably, several amelogenesis-related molecules, including amelogenin, ameloblastin, and enamelin, which are typically expressed during the secretory stage, were significantly downregulated in the molar tooth germs of *Ift*88 mutant mice. Shh signaling, which is crucial for amelogenesis, was also found to be downregulated in the secretory stage of *Ift*88 mutant molars. Intriguingly, the application of an Shh signaling agonist during the secretory stage partially rescued the enamel anomalies observed in *Ift*88 mutant mice [152]. These findings suggest that the function of primary cilia, mediated by IFT88, is indispensable for the secretory stage of amelogenesis, likely through its involvement in Shh signaling [152].

The importance of cilia to heart development can be investigated using a mouse model of cilium-associated cardiovascular abnormalities [130,153]. Mice with heart-specific IFT88 depletion exhibited a spectrum of cardiovascular defects, including double-outlet right ventricle and atrioventricular septal defects [153]. In most specimens, the pulmonary veins were improperly connected to the developing left atrium. Analysis of mutated hearts during early developmental stages revealed abnormal development of the dorsal mesocardium, a second heart-derived structure located at the venous pole that is intrinsically linked to pulmonary vein development [130]. These findings suggest that primary cilia play a critical role in outflow tract development, atrioventricular separation, and the formation of the second cardiogenic structure of the venous pole [130,153].

Research on tissue-specific cilia knockout has significantly advanced our understanding of the tissue-specific functions of cilia, thereby enhancing our knowledge of their importance and facilitating research on ciliopathy treatments [12]. For example, many ciliopathies are associated with congenital heart defects [130], including nephronophthisis, short-rib multi-finger syndrome, Ellis–van Creveld syndrome, Joubert syndrome, Dandy–Walker syndrome, Meckel syndrome, Bardet–Biedl syndrome, and Alstrom syndrome [130]. The study of cilium-related cardiovascular abnormal models holds great promise for the treatment of human cardiovascular diseases and ciliopathies [12,130].

### 3.3. Other Mouse Models

#### 3.3.1. Flexo

*Flexo*, a hypomorphic allele of IFT88, was generated through an ENU mutagenesis screening process. These homozygous mutant mice exhibited polydactyly in all limbs [34], lacked ventral nerve cell types [154], and exhibited other phenotypes characteristic of Hh signaling deficiency.

The Hh family of secreted proteins plays a crucial role in regulating growth and patterning in both invertebrate and vertebrate organisms [155]. In mammalian embryos, Hh proteins are essential for the normal development of numerous organ systems. The loss of Hh function leads to defects in nearly every aspect of mouse embryogenesis, including the absence of left–right asymmetry, dorsalization of the central nervous system, and a significant reduction in digit number [156]. The principal target of Hh signaling is the transcription factor Cubitus interruptus (Ci). In the absence of Hh signaling, Ci is proteolytically processed into a transcriptional repressor, whereas Hh signaling inhibits such processing and allows the unprocessed Ci to act as a transcriptional activator. There are three Ci homologs (Gli1, Gli2, and Gli3) in the mouse. Among these, Gli1 lacks a repressor domain and cannot undergo proteolytic processing, making it an obligate activator [157]. Gli2 and Gli3, however, contain both repressor and activator domains [157].

Despite the absence of ectopic Hh signaling and the downregulation of Hh activity in its normal domain, *flexo* mutant mice develop multiple digits on all four limbs. Similarly, hypomorphic mutants of another mouse IFT gene, *Ift52*, exhibit phenotypes akin to *flexo* mice in multiple organs [34]. These findings suggest that IFT proteins regulate Gli activity, in part, through the proteolytic processing of the Gli3 protein. Thus, IFT protein function is essential for both the activator and repressor activities of the Gli proteins in the Hh pathway [34].

#### 3.3.2. Cobblestone

The hypomorphic allele cobblestone also results from an ENU-induced mutation in the gene encoding IFT88. The advantage of this allele is that embryos survive longer than those with a targeted knockout mutation of IFT88 [99,134,135]. *Cobblestone* mutants exhibit a number of defects, such as severe regionalized defects in the forebrain [99], atrioventricular septal defects in the heart [135], septal defects in the foregut [134], and reduced production of dopaminergic neurons in the midbrain [158].

Researchers have utilized *cobblestone* mutants to illustrate the pivotal role of primary cilia in the development of the dorsal telencephalon. These mutants exhibit severe regionalization defects in the forebrain, characterized by disorganization of the dorsomedial telencephalon, including the choroid plexus, cortical hem, and hippocampus [99].

*Cobblestone* mutants can also be used to study the multiple important roles of primary cilia in heart development [135]. A study revealed a novel connection between Shh signaling at the primary cilium and BMP-dependent effects on cardiogenesis. The results further suggest a potential link between atrioventricular septal defects, the most common congenital heart defects, and genes related to the transport machinery or basal body of the cilia [135].

*Cobblestone* mutants are among the few mouse models that display both correct endodermal dorsoventral specification and defective compartmentalization of the proximal foregut [134]. They serve as exemplary models for tracheoesophageal ciliopathy, offering the opportunity to elucidate the molecular mechanisms by which primary cilia orchestrate the septation process. The numerous malformations observed in *cobblestone* embryos provide deeper insights into a potential link between primary cilia and human VATER/VACTERL syndromes [134].

## 4. Conclusions and Perspectives

Cilia are highly specialized cellular structures that protrude from the cell surface, playing pivotal roles in chemical sensing, osmosis, optical transduction, and motor functions. IFT88 is a crucial protein involved in cilium formation, exhibiting both cilium-related and cilium-independent functions. A deficiency in IFT88 can result in a range of conditions, including organ fibrosis and cyst formation, polydactyly, photoreceptor degeneration, bronchiectasis, and other diseases. Commonly utilized models include ORPK mice and IFT88 conditional allelic mice, which offer valuable tools and avenues for exploring potential treatments for ciliopathies. Research into the IFT88 protein further elucidates how this protein influences cell behavior, development, and disease management through its cilium-related and cilium-independent actions.

## Figures and Tables

**Table 1:** Key mouse models of *Ift88*.

Strain	Alternative Name	Genotype	Construction Method	References
*Ift*88*^Tg^*^737*Rpw*^	ORPK mouse	Hypomorph	Integration of a transgene into an intron adjacent to the 3′ end of the IFT88 gene	Lehman et al. (2008) [131]; Cano et al. (2004) [132]; Coaxum et al. (2014) [133]
*Ift*88*^fl^*	Col3.6cre strain	Tissue-specific knockout	Utilizing the Cre-lox system	Haycraft et al. (2007) [12]; Liu et al. (2004) [129]
*Flexo*	-	Hypomorph	ENU induction	Liu et al. (2005) [34];
*Cobblestone*	-	Hypomorph	ENU induction	Willaredt et al. (2008) [99]; Fitzsimons et al. (2024) [134]; Willaredt et al. (2012) [135]

## Abbreviations

The following abbreviations are used in this manuscript:

ECM	Extracellular matrix
Hh	Hedgehog
IFT	Intraflagellar transport protein
IS	Inner segment
LCA	Leber congenital amaurosis
ORPK	Oak ridge polycystic kidney
OS	Outer segment
PKD	Polycystic kidney disease
RP	Retinitis pigmentosa
